# Different exercise interventions on quality of sleep in breast cancer survivors—a network meta-analysis of randomized controlled trials

**DOI:** 10.3389/fonc.2025.1419613

**Published:** 2025-02-06

**Authors:** Qi Song, You-kang Zhu, Hai Liu, Xiao Liu, Zhang-dong Jiang, Yu-jia Wang, Li-yun Xue, Shao-ying Yang, Xi-fang Liu

**Affiliations:** ^1^ Honghui Hospital, Xi’an Jiaotong University, Xi’an, China; ^2^ School of Physical Education and Health Sciences, Xi’an Physical Education University, Xi’an, China; ^3^ Xi'an Jiaotong University First Affiliated Hospital, Xi’an Jiaotong University, Xi’an, China

**Keywords:** exercise, sleep quality, breast cancer, psycho-oncology, network meta-analysis, walking training, systematic review

## Abstract

**Introduction:**

Breast cancer is currently the most prevalent cancer globally; however, it generally has a favorable prognosis and is linked to a high survival rate. While effective treatments can extend survival and mitigate associated side effects, not all survivors are exempt from complications. Notably, a significant proportion of survivors experience sleep disorders following surgery, which can severely impact their quality of life. Exercise is frequently recommended as a non-pharmacological intervention to enhance sleep quality among breast cancer survivors and may also play a role in reducing recurrence rates. Recognizing that various forms of exercise may yield different outcomes in addressing sleep disorders in this population, we conducted a network review meta-analysis to assess the effectiveness of diverse exercise modalities for breast cancer survivors suffering from sleep disturbances.

**Methods:**

We searched four electronic databases for randomized controlled trials of individuals diagnosed breast cancer with sleep disorders by different exercise therapy. The primary outcomes included Yoga, Pilates, Qigong, Tai Chi, Walking, Dance, Resistance training, Football, Virtual reality therapy, Activity change exercise, Software-guided exercises. The methodological quality of the included studies was assessed using the Cochrane Bias risk Assessment tool, and network meta-analysis was performed using Stata15 software. The review was pre-registered (PROSPERO ID: CRD42023442892).

**Results:**

Data on 3083 breast cancer survivors with sleep disturbances from 34 eligible randomized controlled trials were analyzed, with 23 classified as medium risk and 2 as high risk. Network meta-analysis showed that walking exercise [Standard Median Different (SMD) =3.06, 95% Confidence Interval (95%CI)=(-5.89,-0.23)] significantly improved sleep disorder (Surface Under the Cumulative Ranking curve, SUCRA: 84.5%) and reduced Pittsburgh sleep quality index (PSQI) score.

**Discussion:**

Based on the network ranking table, we can conclude that walking exercise offers greater benefits compared to other exercise interventions for improving sleep quality in breast cancer patients. This finding presents a novel perspective on exercise interventions for breast cancer survivors experiencing sleep disorders.

**Systematic review registration:**

PROSPERO https://www.crd.york.ac.uk/PROSPERO/display_record.php?RecordID=442892, identifier CRD42023442892.

## 1 Introduction

According to the International Agency for Research on Cancer (IARC) of the World Health Organization, there were 10.29 million new cancer cases worldwide in 2020. Among these, breast cancer accounted for 2.26 million cases, making it the most commonly diagnosed cancer in women and surpassing lung cancer as the highest incidence of cancer globally (1). Despite the increasing incidence of breast cancer, the widespread availability of health screening and advancements in treatment have led to a remarkable 5-year survival rate of 90% for breast cancer patients (2). This significant improvement not only reduces the mortality rate among breast cancer patients but also contributes to a growing population of breast cancer survivors (BCS). However, BCS do not experience a complete absence of symptoms; rather, negative psychological factors and side effects—including pain, anxiety, depression, cancer-related fatigue, and sleep disturbances—significantly affect the overall quality of life of survivors (3). Therefore, it is crucial to give due consideration and provide appropriate treatment for the long-term side effects.

Breast cancer survivors often experience high rates of sleep disorders (4–7), which persist long after treatment and can exacerbate inflammation and physiological discomfort through their impact on the stress response system. While drug treatments are the go-to solution, their uncertain efficacy and potential side effects have spurred a search for safer, alternative therapies to enhance survivors’ quality of life and prognosis.

Exercise is often recommended as a non-drug treatment for sleep disorders in cancer survivors, offering benefits in life quality and potentially lowering breast cancer recurrence. Studies like those by Kelsey L. Sinclair (8) and Jingwen Liao (9) have shown positive impacts of yoga and Baduanjin on sleep and mood, while Jayani Sagaz Hiansdt’s (10) research found mixed dance had no clear effect. Given unclear guidelines and limited data on exercise for sleep improvement in breast cancer patients, our network meta-analysis compares different exercises’ effects on BCS sleep quality to suggest optimal exercise regimens. The study aims to guide exercise prescriptions for BCS with sleep issues.

Network meta-analysis, an advanced evidence-based method, has benefits beyond traditional meta-analysis by analyzing complex variable relationships and ranking different interventions’ significance in a network. It quantitatively compares various treatments for the same condition, helping to determine the most effective based on outcomes.

This study deployed network meta-analysis to examine the effects of different exercise regimens (like Yoga, Pilates, Qigong, Tai Chi, etc.) on sleep quality in breast cancer patients. The results bolster theoretical support for exercise therapy in managing sleep disorders for breast cancer survivors and offer evidence-based exercise medical guidance for patients and healthcare providers.

## 2 Materials and methods

The implementation and reporting of this review were based on an expanded statement of the Treatment network meta-analysis of the Preferred Reports Program for Systematic Evaluation and Analysis (PRISMA). This review established a prior protocol and registered with the International Prospective Systems Evaluation Registry (PROSPERO). bearing the registration number CRD42023442892.

### 2.1 Study sources and search strategy

We systematically used four electronic databases (PubMed, EMBASE, Cochrane Central Register of Controlled Trials, and Web of Science) to conduct a comprehensive search following PRISMA guidelines using keywords and free words. The time limit of the search was from the establishment of the database to May 8, 2023, with no restrictions on language and date. The search strategy is strictly built according to the PICOS tool, namely: (P) Population: BCS; (I) Intervention: different forms of movement; (C) Comparator: the control group receiving only conventional care and the experimental group receiving appropriate exercise rehabilitation measures; (O) Outcomes: Assessment of sleep quality in BCS; (S) Study Type: randomized controlled trial. The detailed search strategy is shown in **Table 1** (taking Pubmed as an example).

**Table 1:** The detailed search strategy.

#1	(((((((((((((((((((((((((exercise[MeSH Terms]) OR (Exercises[Title/Abstract])) OR (Physical Activity[Title/Abstract])) OR (Activities, Physical[Title/Abstract])) OR (Activity, Physical[Title/Abstract])) OR (Physical Activities[Title/Abstract])) OR (Exercise, Physical[Title/Abstract])) OR (Exercises, Physical[Title/Abstract])) OR (Physical Exercise[Title/Abstract])) OR (Physical Exercises[Title/Abstract])) OR (Acute Exercise[Title/Abstract])) OR (Acute Exercises[Title/Abstract])) OR (Exercise, Acute[Title/Abstract])) OR (Exercises, Acute[Title/Abstract])) OR (Exercise, Isometric[Title/Abstract])) OR (Exercises, Isometric[Title/Abstract])) OR (Isometric Exercises[Title/Abstract])) OR (Isometric Exercise[Title/Abstract])) OR (Exercise, Aerobic[Title/Abstract])) OR (Aerobic Exercise[Title/Abstract])) OR (Aerobic Exercises[Title/Abstract])) OR (Exercises, Aerobic[Title/Abstract])) OR (Exercise Training[Title/Abstract])) OR (Exercise Trainings[Title/Abstract])) OR (Training, Exercise[Title/Abstract])) OR (Trainings, Exercise[Title/Abstract])
#2	(((Sleep quality) OR (Qualities, Sleep)) OR (Quality, Sleep)) OR (Sleep Qualities)
#3	(((((((((((((((((((((((((exercise[MeSH Terms]) OR (Exercises[Title/Abstract])) OR (Physical Activity[Title/Abstract])) OR (Activities, Physical[Title/Abstract])) OR (Activity, Physical[Title/Abstract])) OR (Physical Activities[Title/Abstract])) OR (Exercise, Physical[Title/Abstract])) OR (Exercises, Physical[Title/Abstract])) OR (Physical Exercise[Title/Abstract])) OR (Physical Exercises[Title/Abstract])) OR (Acute Exercise[Title/Abstract])) OR (Acute Exercises[Title/Abstract])) OR (Exercise, Acute[Title/Abstract])) OR (Exercises, Acute[Title/Abstract])) OR (Exercise, Isometric[Title/Abstract])) OR (Exercises, Isometric[Title/Abstract])) OR (Isometric Exercises[Title/Abstract])) OR (Isometric Exercise[Title/Abstract])) OR (Exercise, Aerobic[Title/Abstract])) OR (Aerobic Exercise[Title/Abstract])) OR (Aerobic Exercises[Title/Abstract])) OR (Exercises, Aerobic[Title/Abstract])) OR (Exercise Training[Title/Abstract])) OR (Exercise Trainings[Title/Abstract])) OR (Training, Exercise[Title/Abstract])) OR (Trainings, Exercise[Title/Abstract])
#4	#1 AND #2 AND #3
#5	((((((((((((((Aquatic Therapy[MeSH Terms]) OR (Therapy, Aquatic)) OR (Aquatic Exercise)) OR (Aquatic Exercise Therapy)) OR (Exercise Therapy, Aquatic)) OR (Therapy, Aquatic Exercise)) OR (Water Exercise Therapy)) OR (Exercise Therapy, Water)) OR (Therapy, Water Exercise)) OR (Pool Therapy)) OR (Therapy, Pool)) OR (Ai Chi Therapy)) OR (Therapies, Ai Chi)) OR (Therapy, Ai Chi)) OR (Water Tai Chi Therapy)
#6	#1 AND #2 AND #5
#7	Bicycling[All Fields]
#8	#1 AND #2 AND #7
#9	(Walking[All Fields]) OR (Ambulation[All Fields])
#10	#1 AND #2 AND #9
#11	(running[MeSH Terms]) OR (runnings[All Fields])
#12	#1 AND #2 AND #11
#13	Yoga[All Fields]
#14	#1 AND #2 AND #13
#15	((((((((((Tai JiMeSH Terms]) OR (Tai-i) OR (TaiChi) OR (Chi Tal)) OR (Tal Ji Quan)) OR (J Quan,Tal)) OR (QuanTalJ)) OR (Tal) OR (Taljiquan)) OR (T’al Chi)) OR (Tai Chi Chuan)
#16	#1 AND #2 AND #15
#17	(baduanjin[All Fields]) OR (baduanjin exercise[All Fields])
#18	#1 AND #2 AND #17
#19	((((((((((Dancing[MeSH Terms) OR (Dance Therapy[MeSH Terms])) OR (Dance)) OR (Ballet)) OR (Square Dance)) OR (Dance, Square)) OR (Hip-Hop Dance)) OR (Dance Hip-Hop)) OR (Hip Hop Dance) OR (Jazz Dance)) OR (Dance, Jazz)) OR (Tap Dance)) OR (Dance, Tap)) OR (Modern Dance)) OR (Dance,Modern)) OR (Salsa Dancing)) OR (Dancing. Salsa)) OR (Line Dancing)) OR (Dancing Line)) OR (Therapy. Dance)) OR (Dance Therapies)) OR (Therapies, Dance)
#20	#1 AND #2 AND #19
#21	((((((((((((Exergaming[MeSH Terms]) OR (Exergamings))OR (Active-Video Gaming)) OR (Active Video Gaming)) OR (Active-Video Gamings)) OR (Gaming.Active-Video)) OR (Gamings,Active-Video)) OR (Virtual Reality Exercise)) OR (Exercise, Virtual Reality)) OR (Exercises. Virtual Reality)) OR (Virtual Reality Exercises)) OR (Exergames)) OR (Exergame)
#22	#1 AND #2 AND #21
#23	(((((((((((((((((((((((Resistance Training[MeSH Terms]) OR (Training, Resistance)) OR (Strength Training)) OR (Training, Strength)) OR (Weight-Lifting Strengthening Program)) OR (Strengthening Program, Weight-Lifting)) OR (Strengthening Programs, Weight-Lifting)) OR (Weight Lifting Strengthening Program)) OR (Weight-Lifting Strengthening Programs)) OR (Weight-Lifting Exercise Program)) OR (Exercise Program, Weight-Lifting)) OR (Exercise Programs, Weight-Lifting)) OR (Weight Lifting Exercise Program)) OR (Weight-Lifting Exercise Programs)) OR (Weight-Bearing Strengthening Program)) OR (Strengthening Program, Weight-Bearing)) OR (Strengthening Programs, Weight-Bearing)) OR (Weight Bearing Strengthening Program)) OR (Weight-Bearing Strengthening Programs)) OR (Weight-Bearing Exercise Program)) OR (Exercise Program, Weight-Bearing)) OR (Exercise Programs, Weight-Bearing)) OR (Weight Bearing Exercise Program)) OR (Weight-Bearing Exercise Programs)
#24	#1 AND #2 AND #23
#25	(((((((Exercise Movement Techniques[MeSH Terms]) OR (Movement Techniques, Exercise)) OR (Exercise Movement Technics)) OR (Pilates-Based Exercises)) OR (Exercises, Pilates-Based)) OR (Pilates Based Exercises)) OR (Pilates Training)) OR (Training, Pilates)
#26	#1 AND #2 AND #25
#27	whole-body vibration training
#28	#1 AND #2 AND #27
#29	((QIGONG) OR (Qi Gong)) OR (Ch’i Kung)
#30	#1 AND #2 AND #29
#31	((Football) OR (Football, American)) OR (American Football)
#32	#1 AND #2 AND #31
#33	(Gymnastics) OR (Calisthenics)
#34	#1 AND #2 AND #33
#35	((((Soccer) OR (Football European)) OR (European, Football)) OR (Europeans, Football)) OR (European Football)) OR (Football, European)
#36	#1 AND #2 AND #35
#37	(((Track and Field) OR (Field and Track)) OR (Track)) OR (Tracks)
#38	#1 AND #2 AND #37

### 2.2 Inclusion and exclusion criteria

Studies were selected based on the following inclusion criteria for eligibility:

Subjects: Survivors of breast cancer surgery with sleep disturbances; (2) Study type: Clinical randomized controlled trial; (3) Intervention measures: Based on conventional nursing, the experimental group used different exercise methods for BCS’ intervention (any time, place, frequency, intensity and duration cycle), while the control group used conventional nursing; (4) Outcome indicators: including at least one of the following scales: Pittsburgh Sleep Quality Index(PSQI), Insomnia Severity Index(ISI), Medical Outcomes Study Sleep Scale(MOS-SS), The M. D. Anderson Symptom Inventory (MDASI), or subjective measures of sleep status such as Actual sleep time or Number of awakenings during the night.

Exclusion criteria were:

(1) Repeated publication and repeated literature; (2) Incomplete data, data format does not conform to, data cannot be converted; (3) The full text cannot be obtained; (4) Patients with other cancers; (5) Sleep quality was not evaluated when the quality of life was used as an outcome indicator; (6) Non-clinical randomized controlled trials (such as cross-sectional studies, animal experiments, conference abstracts, monographs, reviews, meta-analyses, and other secondary analyses or clinical guidelines).

### 2.3 Study selection

The files retrieved from different databases were imported into EndNote X9 and merged and de-duplicated. Two researchers independently read the title and abstract of the literature. They conducted a preliminary screening according to inclusion and exclusion criteria, excluding nonrandomized controlled trials, cross-sectional studies, conference minutes, reviews, Meta, and other irrelevant literature. If reading the title and abstract cannot determine whether to include the study, screening is performed by reading the full text. After the two researchers finished the screening, they cross-checked and compared the remaining literature. If they were identical, they were directly included. In case of differences, they discussed and negotiated in the form of discussion.

### 2.4 Data extraction

This step was independently implemented by two reviewers. Available data were extracted from each study using a standardized, pre-selected data extraction table under the following headings: (1) first author; (2) the state; (3) Publication time; (4) Research object; (5) Treatment method; (6) Sample size; (7) Average age; (8) The intervention measures of the experimental group and control group: exercise mode, cycle, time and frequency; (9) Outcome indicators. Two reviewers compared completed pre-designed forms. When no consensus is reached after discussion, differences are discussed by two and third reviewers and finally agreed upon by three parties.

### 2.5 Risk of bias of individual studies

Two researchers independently assessed the risk of bias (ROB) using tools in ROB2_IRPG_beta_v9. We considered the following seven areas: (1) random sequence generation, (2) covert treatment allocation, (3) blinding of participants, (4) personnel, (5) incomplete outcome data, (6) selective reporting, and (7) other sources of bias. Trials were classified into three ROB levels based on the number of possible high ROB ingredients: high risk (5 or more), moderate risk (4), and low risk (3 or less). The two researchers documented their results in an evaluation form and reviewed them after the evaluation was completed. In case of any disagreement, they resolved the issue through discussion. If the disagreement could not be resolved through discussion, they sought the input of a third reviewer and continued the discussion until a consensus was reached among all three parties. The bias risk assessment results were evaluated using Review Manager 5.3.

### 2.6 Study design

This study was designed to evaluate the effects of various exercise interventions compared to a non-exercise control group on the quality of sleep in breast cancer survivors. The focus was on direct comparisons to determine the specific impact of each exercise intervention.

### 2.7 Statistical analysis

Meta-analysis was performed using Revman 5. software. All variables were continuous and the mean value was represented as standard deviation (S.D.). 95% confidence intervals (CI) and individual standardized mean differences (SMD) were used. Alternatively, standardized mean differences (SMD) will be used for data consolidation in trials with different scales, which is the mean difference between the results of the groups divided by the standard deviation of the results between the subjects (11). If the outcome indicators in the included literature were not in the form of mean and standard deviation, they were transformed into mean and standard deviation through formula calculation for statistical analysis.

Stata software was used to generate network of evidence and funnel graphs to compare and rank different exercise interventions. The consistency test was conducted initially, and due to potential differences between studies, the random effects model was chosen for analysis instead of the fixed effects model. Following the PRISMA NMA specification, NMA aggregation and analysis were performed using Markov Monte Carlo simulation chains within a Bayes-based framework. To assess the consistency between indirect and direct comparisons, the node method was employed, and calculations were done using the instructions provided in the Stata software. If the P value is greater than 0.05, it indicates that the consistency test is passed, and the consistency model is adopted. The network evidence graph was utilized to visually represent and describe the relationship between exercise interventions and the control group. Each node represented a different exercise intervention or control condition, and the lines connecting the nodes indicated favorable comparisons between the interventions. The size of each node and the width of the connecting lines were proportional to the number of studies.

Intervention grades were summarized and reported as P scores. The P-score represents the frequency simulation of the area under the cumulative sequencing curve (surface under the cumulative ranking, SUCRA) value. We calculated the certainty (averaging) across all competing treatments. The P score ranges from 0 to 1, where 1 indicates the best treatment without uncertainty, and 0 indicates the worst treatment with no uncertainty. While P scores or SUCRA can be represented as a percentage of effectiveness or acceptability of exercise interventions, it is important to interpret these scores cautiously unless there are clinically meaningful differences between interventions. To assess if small-scale studies introduce a bias that could lead to NMA publication bias, we created a network funnel plot, which was visually inspected for symmetry.

## 3 Results

### 3.1 Study, identification, and selection

**Figure 1:**
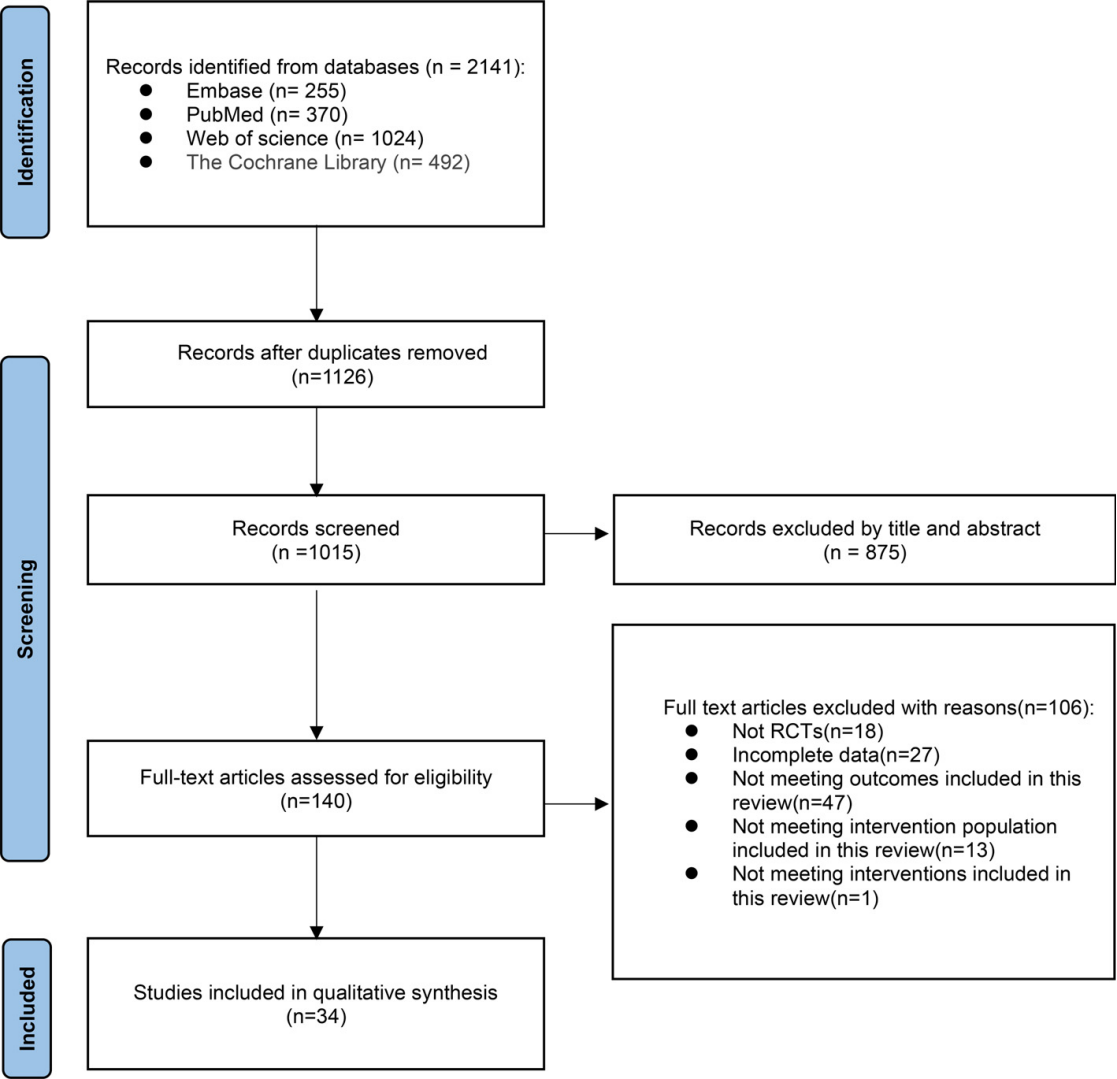
Preferred reporting items for systematic reviews and meta-analysis (PRISMA) flow diagram.

The initial search in databases yielded a total of 2141 articles, including PubMed (n=370) and Embase (n=255), Web of Science (n=1024), and Cochrane Library (n=492). After eliminating duplication, the remaining 1015 articles were read with titles and abstracts, and 875 were deleted. After full-text reading of the remaining 140 literature, 105 literature were excluded for the following reasons: non-randomized controlled study (n=18), incomplete data (n=27), failure to meet the outcome indicators of this study (n=47), failure to fully meet the intervention population of this study (n=12), and failure to meet the intervention measures included in this study (n=1). Finally,34 RCT studies were confirmed and included in the analysis (8–10, 12–42) (**Figure 1**).

### 3.2 Study characteristics included in the study

**Figure 2:**
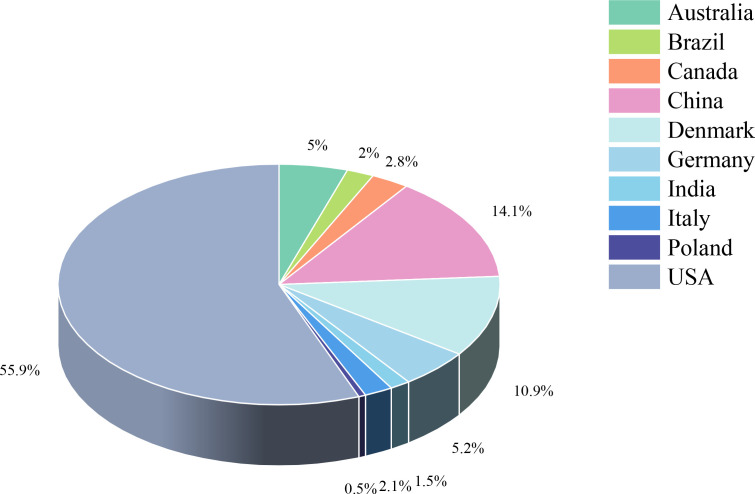
Study characteristics.

**Figure 3:**
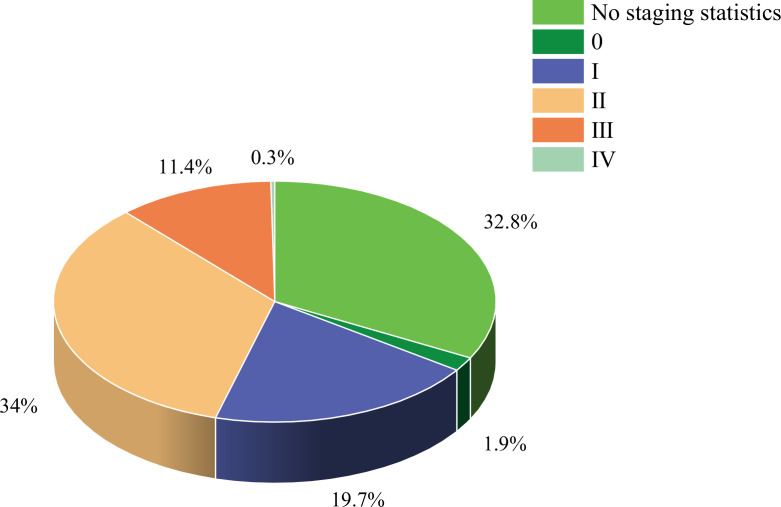
Study characteristics.

The study included 34 randomized controlled trials involving 3083 BCS with sleep disturbances (8–10, 12–42). Interventions in the experimental group included Yoga (8, 13, 18, 21–25, 28–30, 32, 33, 37–39, 42), Pilates (14), Qigong (9, 31, 34), Tai Chi (12, 34, 41), Walking (17, 19), Dance (10, 15), Resistance training (27), Football (20), Virtual reality therapy (40), Activity change exercise (26, 35, 36), and Software-guided exercises (16). In addition to the conventional information provided in the literature, this study also assessed the proportion of nationalities among BCS and the distribution of cancer stages. Among the subjects included, the United States had the highest proportion of survivors at 55.9%, while Poland had the lowest at 0.5%. The proportions for China, Denmark, Germany, Australia, Canada, Italy, Brazil, and India decreased sequentially. Regarding cancer stages, patients were distributed as follows: stage 0 accounted for 1.9%, stage I for 19.7%, stage II for 34%, stage III for 11.4%, and stage IV for 0.3%. In addition, the clinical stage of breast cancer was not specified for an additional 32.8% of the breast cancer survivors. (**Figures 2**, **3**, **Table 2**).

**Table 2:** Study characteristics.

Author	Country	Year	Population	Treatment	Age (mean+SD)	Total/male/female	Intervention	Control	Outcome
Oliver Czech	Poland	2023	Breast Cancer No staging statistics.	No staging statistics.	T: 50.59 (12.64) C: 59.55 (7.85)	T:9/0/9 C:7/0/7	Virtual Therapeutic Garden therapy sessions Length of Intervention: 2 weeks Freq: 7 times a week Duration: 15 min	CON	PSQI
Jingwen Liao	China	2022	Breast Cancer Disease Stage I II III T: 6 20 7 C: 11 16 8	T C Aromatase inhibitor:33 35	T: 53.12 (7.02) C: 54.63 (8.44)	T: 33/0/33 C: 35/0/35	Baduanjin Length of Intervention: 12 weeks Freq: 2 times a week Duration: 90 min	CON	PSQI
Li-Qun Yao	Australia	2022	Breast Cancer Disease Stage I II III T: 2 29 5 C: 7 21 8	T C Surgery:36 36 Chemotherapy:36 36	T: 45.3(8.5) C: 48.7(7.8)	T: 36/0/36 C: 36/0/36	Tai chi Length of Intervention: 8 weeks Freq: 2 times a week Duration: 50 min	CON	PSQI
KelseyL. Sinclair	USA	2022	Breast Cancer Disease Stage I II III T: 18 44 17 C: 19 52 24	T C Chemotherapy:79 95	T: 46.96(9.83) C:48.95(10.32)	T: 79/0/79 C: 95/0/95	Yoga Length of Intervention: 4-12weeks Freq: 1 class per week or every 3 weeks Duration: 75-90 min	CON	PSQI
Laura Q. Rogers	USA	2023	Breast Cancer Disease Stage All: I-IIIA	No staging statistics.	T: NA C: NA	T: 101/0/101 C: 100/0/100	Exercise Length of Intervention: 12 weeks Freq: 3 times a week Duration: NA	CON	PSQI
Jayani Sagaz Hiansdt	Brazil	2021	Breast Cancer No staging statistics.	T C Chemotherapy:1 1 Radiotherapy:1 0 Hormone:5 9 Surgery:11 10	T: 55.7 (7.3) C: 54.8 (9.6)	T: 11/0/11 C: 10/0/10	Dance Length of Intervention: 12 weeks Freq: 2 times a week Duration: 60 min	CON	PSQI
Nga H. Nguyen	Australia	2021	Breast Cancer No staging statistics.	No staging statistics.	T+C:62(6.4)	T: 43/0/43 C: 40/0/40	A wearable technology-based physical activity Length of Intervention: 24 weeks Freq: 7 nights a week Duration: NA	CON	PSQI
Li Wang	China	2020	Breast Cancer Disease Stage I II III T: 4 31 9 C: 3 34 7	T C Surgery:44 44	T+C:48.2(9.7)	T: 44/0/44 C: 44/0/44	Yoga Length of Intervention: 6 weeks Freq: 7 times a week Duration: 120-180 min	CON	PSQI
Yu Zhang	China	2020	Breast Cancer No staging statistics.	T C Chemotherapy:30 30 Surgery:30 30	T: NA C: NA	T: 30/0/30 C: 30/0/30	Football Length of Intervention: 24 weeks Freq: 3 times a week Duration: 60-120 min	CON	PSQI
Alejandro Chaoul	USA	2018	Breast Cancer Disease Cancer I II III T: 18 39 17 C: 16 48 21	T C Chemotherapy:55 72 Surgery:74 85	T: 49.5(9.8) C: 49(10.1)	T: 74/0/74 C: 85/0/85	Yoga Length of Intervention: 12 weeks Freq: NA Duration: 75–90 min	CON	PSQI
Rainbow T.H. Ho	China	2016	Breast Cancer Disease Stage 0 I II III T: 5 17 27 18 C: 4 18 31 15	T C Surgery:67 68 Chemotherapy:13 16	T+C: 48.9(8.2)	T: 67/0/67 C: 68/0/68	Dance Length of Intervention: 3 weeks Freq: 2 times a week Duration: 90 min	CON	PSQI
Chelsea G. Ratcliff	USA	2016	Breast Cancer Disease Stage 0 I II III T: 5 16 15 17 C: 7 17 15 15	T C Surgery:53 54 Chemotherapy:36 34	T: 52.38(9.82) C: 52.11(9.85)	T: 53/0/53 C: 54/0/54	Yoga Length of Intervention: 6 weeks Freq: 3 times a week Duration: 60 min	CON	PSQI
Linda K. Larkey	USA	2015	Breast Cancer Disease Stage I II III T: 20 22 1 C: 14 18 2	No staging statistics.	T: 57.4(8.94) C: 59.8(8.93)	T:43/0/43 C: 34/0/34	QiGong Length of Intervention: 12 weeks Freq: 5 times a week Duration: 30 min	CON	PSQI
Shelley A. Johns	USA	2015	Breast Cancer Disease Stage 0 I II III IV T: 2 5 5 4 2 C: 0 7 7 2 1	T C Radiation:10 12 Chemotherapy:11 12 Chemotherapy& Radiation: 7 8 Endocrine: 12 8	T: 58.8(9.3) C: 55.7(9.3)	T: 18/0/18 C: 17/0/17	Yoga Length of Intervention: 7 weeks Freq: 7 times a week Duration: 120 min	CON	ISI
Signe R. Andersen	Denmark	2013	Breast Cancer Disease Stage I II III T: 51 109 8 C :64 101 3	T C Chemotherapy:78 82 Radiotherapy: 125 146 hormonal therapy:91 88	T: 53.9(10.1) C: 54.4(10.5)	T: 168/0/168 C: 168/0/168	Yoga Length of Intervention: 41 weeks Freq: 7 times a week Duration: 45 min	CON	MOS-SS
Zhen Chen	China	2013	Breast Cancer Disease Stage 0 I II III T:2 8 17 17 C:3 14 17 7	T C Surgery:44 41 Radiotherapy: 39 35	T: 45.3(6.3) C: 44.7(9.7)	T: 44/0/44 C: 41//0/41	QiGong Length of Intervention: 5-6 weeks Freq: 5 times a week Duration: 40 min	CON	PSQI
Julienne E. Bower, Ph.D	USA	2012	Breast Cancer Disease Stage All:0-II	T C Chemotherapy:8 9 Radiotherapy: 11 13 Inhibitor:12 10	T: 54.4(5.7) C: 53.3(4.9)	T: 16/0/16 C: 15/0/15	Yoga Length of Intervention: 12 weeks Freq: 2 times a week Duration: 90 min	CON	PSQI
Cecile A. Lengacher	USA	2012	Breast Cancer Disease Stage 0 I II III T:5 26 7 3 C:9 19 12 3	T C Radiotherapy:25 26 Radiation & Chemotherapy:16 17	T+C: 58(9.4)	T: 41/0/41 C: 43/0/43	Yoga Length of Intervention: 6 weeks Freq: 7 times a week Duration: 120 min	CON	MDASI
Ya-Jung Wang	USA	2011	Breast Cancer Disease Stage I II III T:9 26 0 C:7 30 0	T C Surgery:35 37 Radiotherapy: 15 17 Reconstruction:5 4 Chemotherapy:35 37	T:48.4(10.15) C: 52.3 (8.84)	T: 35/0/35 C: 37/0/37	Walking Length of Intervention: 6 weeks Freq: 3-5 times a week Duration: 30-50 min	CON	PSQI
Kavita D. Chandwani	USA	2010	Breast Cancer Disease Stage I II III T: 5 12 10 C:10 15 4	T C Surgery:27 29 Chemotherapy:23 24	T: 51.39 (7.97) C: 54.02(9.96)	T: 27/0/27 C: 29/0/29	Yoga Length of Intervention: 6 weeks Freq: 2 times a week Duration: 60 min	CON	PSQI
Kavita, D. Chandwani	USA	2014	Breast Cancer Disease Stage 0 I II III T:5 16 15 17 C:7 17 15 15	T C Surgery:53 54 Chemotherapy:36 34	T: 52.38(1.35) C: 52.11(1.34)	T: 53/0/53 C: 54/0/54	Yoga Length of Intervention: 6 weeks Freq: 3 times a week Duration: 60 min	CON	PSQI
Janice K. Kiecolt-Glaser,	USA	2014	Breast Cancer Disease Stage 0 I II III T:8 45 36 7 C:7 38 36 9	T C Surgery:13 13 Chemotherapy:23 23 Radiation:24 14 Radiation & Chemotherapy:36 40	T: 51.8(9.8) C: 51.3(8.7)	T: 96/0/96 C: 90/0/90	Yoga Length of Intervention: 12 weeks Freq: 2 times a week Duration: 90 min	CON	PSQI
Teletia R. Taylor	USA	2018	Breast Cancer No staging statistics.	T C Surgery:9 11	T: 54.9(8.8) C: 52.6(8.2)	T: 9/0/9 C: 11/0/11	Yoga Length of Intervention: 8 weeks Freq: 1 times a week Duration: 75 min	CON	ISI
Eliana Roveda	Italy	2017	Breast Cancer No staging statistics.	T C Only Surgery:19 21	T:55.2(6.8) C: 58.2(6.4)	T: 19/0/19 C: 21/0/21	Walking Length of Intervention: 12 weeks Freq: 2 times a week Duration: 70 min	CON	Actual sleep time
Karen Steindorf	Germany	2017	Breast Cancer No staging statistics.	T C Surgery: 80 80 Radiation boost:60 59 Radiation technology:63 57 Chemotherapy:68 71 Hormone:43 35	T: 55(9.4) C: 56.2(8.6)	T: 80/0/80 C: 80/0/80	machine-based progressive resistance exercises Length of Intervention: 12 weeks Freq: 2 times a week Duration: 3 sets,8–12 repetitions at 60–80% 1RM	CON	Number of awakenings during the night
Suzanne C. Danhauer	USA	2009	Breast Cancer Disease Stage I II III T:8 10 4 C:14 3 5	T C Radiation:6 3 Chemotherapy:8 3	T: 54.3 (9.6) C:57.2 (10.2)	T: 22/0/22 C: 22/0/22	Yoga Length of Intervention: 10 weeks Freq: 10 times a week Duration: 75 min	CON	PSQI
Judith K. Payne	USA	2008	Breast Cancer No staging statistics.	T C Hormonal treatment:.10 10	T+C: 64.7(6.3)	T: 10/0/10 C: 10/0/10	Walking Length of Intervention: 14 weeks Freq: 4 times a week Duration: 20 min	CON	PSQI
Simona Micheletti	Italy	2022	Breast Cancer Disease Stage 0 I II III T: 1 1 6 4 C:0 2 7 3	T C Radiotherapy:12 12	T: 47(15) C: 53(9)	T: 12/0/12 C: 12/0/12	yoga Length of Intervention: 5 weeks Freq: 5 times a week Duration: 75 min	CON	PSQI
Keyla de Paula Barbosa	Brazil	2021	Breast Cancer Disease Stage I II III T:4 14 2 C5 14 1	T C surgery:20 20 Chemotherapy: 20 20 Radiation therapy:13 12	T: 52.0(14.25) C: 49.8(13)	T: 20/0/20 C: 20/0/20	Pilates Length of Intervention: 8weeks Freq: 2 times a week Duration: 75 min	CON	PSQI
Raghavendra Mohan Rao	India	2017	Breast Cancer No staging statistics.	T C Surgery& Chemotherapy& Radiation: 8 7 Chemotherapy& Radiation: 11 17 Surgery& Chemotherapy& Radiation& Chemotherapy:26 22	T: 48.9(9.1) C: 50.2(9.2)	T: 25/0/25 C: 21/0/21	Yoga Length of Intervention: 12 weeks Freq: at least 2 times a week Duration: 60 min	CON	PSQI
Isabelle Bragard	Canada	2017	Breast Cancer Disease Stage 0 I II III T:1 12 7 1 C:1 37 22 5	T C Radiation:10 30 Hormonal:12 35	T: 54(11) C: 54.3(10)	T: 21/0/21 C:65/0/65	Yoga Length of Intervention: 1 weeks Freq: 6 times a week Duration: 90 min	CON	ISI
Michael R	USA	2017	Breast Cancer No staging statistics.	T C Surgery:6 4 Chemotherapy:7 4 Radiation:11 17 Chemotherapy & radiation: 11 17	T: 59.6(7.9) C: 60.0(9.3)	T: 45/0/45 C: 45/0/45	TaiJi Length of Intervention: 60 weeks Freq: 120 sessions a week	CON	PSQI
Suzanne C Danhauer	USA	2015	Breast Cancer Disease Stage I II III T:3 10 8 C:4 6 7	T C surgery:21 17	T: 50(29-83) C: 45(30-65)	T: 21/0/21 C: 17/0/17	Yoga Length of Intervention: 10 weeks Freq: 8-10 times a week Duration: 75 min	CON	MOS-SS

### 3.3 Quality assessment of the included studies types of exercise treatment

**Figure 4:**
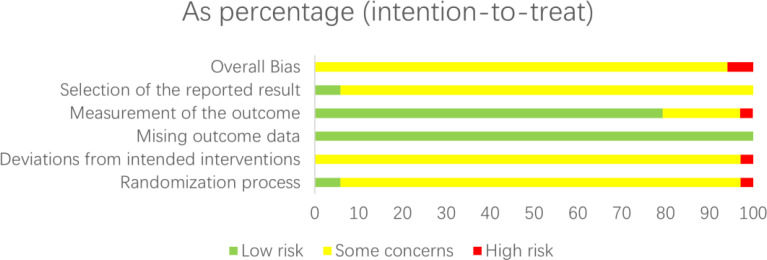
Risk of bias graph: review authors’ judgements about each risk of bias item presented as percentages across all included studies.

**Figure 5:**
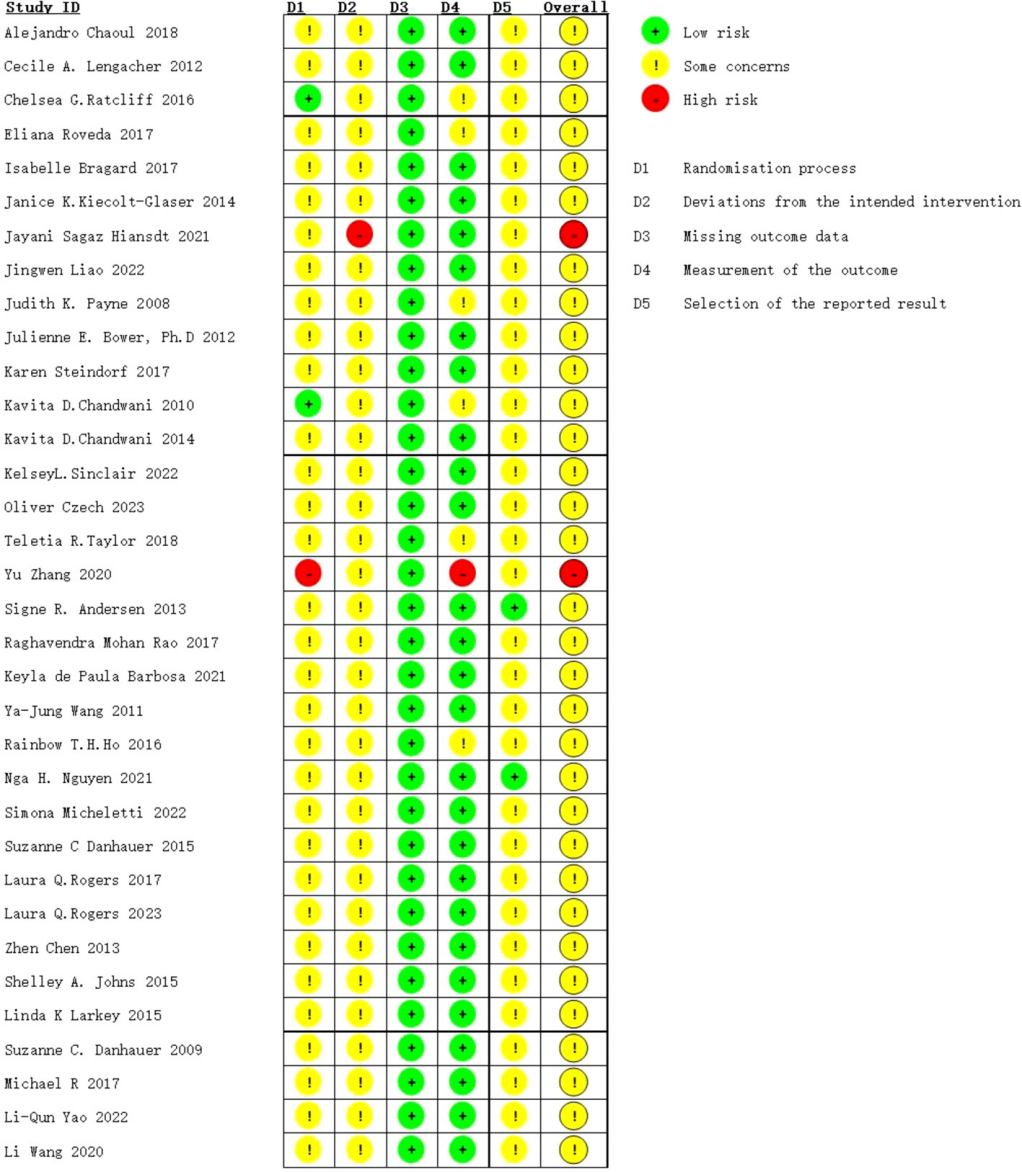
Risk of bias summary: review authors’ judgements about each risk of bias item for each included study.

Risk of bias (ROB) was assessed independently using the ROB2_IRPG_beta_v9 tool. Two researchers evaluated the quality of 34 articles (8–10, 12–42), with 23 classified as medium risk (8, 9, 12–19, 21–42) and 2 as high risk (10, 20). Therefore, the literature included in this study was generally of aboveaverage quality (**Figures 4**, **5**).

### 3.4 The interventions of exercise

A total of 11 interventions for BCS with sleep disorder were identified, including: 17 Yoga (8, 13, 18, 21–25, 28–30, 32, 33, 37–39, 42), 3 Activity change exercise (26, 35, 36),2 Walking (17, 19), 3 Tai Chi (12, 34, 41) and Qigong exercises (9, 31, 34), 2 Dance (10, 15), and 1 Pilates (14), Virtual reality therapy (40), Football (20), Resistance training (27), and Software-guided exercises (16). Thirty-one studies reported the time, frequency, and total duration of each exercise (8–10, 12–15, 17–29, 31–34, 36–42), while two studies did not report the time of each exercise (16, 35), and one study did not report the frequency of exercise (30). The intervention was administered 1 to 7 times per week for a duration of 2 to 41 weeks. The duration of each intervention varied between 15 and 180 minutes, depending on the patient. The control group received either usual care or regular visits.

### 3.5 Sleep quality assessments

Different sleep assessment methods were used in the included literature. The Pittsburgh Sleep Quality Index (PSQI) was used as an outcome indicator in 26 studies (8–10, 12, 14–20, 24–26, 28–32, 34, 35, 38–42), while the Insomnia Severity Index (ISI) was used in three studies (21, 33, 37). The Medical Outcomes Study Sleep Scale (MOS-SS) was used in two studies (13, 22), and one study used the Number of Awakenings during the night as the outcome index (27). Additionally, one study used actual sleep time (36), and another study used the MD Anderson Symptom Inventory (MDASI) as an outcome measure (23). PSQI is a widely used tool for assessing sleep quality. It is a self-reported questionnaire that is administered monthly. The questionnaire consists of 19 items, which assess seven components of subjective sleep quality, sleep latency, sleep duration, sleep efficiency, sleep disorders, drug use, and daytime dysfunction. PSQI scores range from 0 to 21, with higher scores indicating poorer sleep quality. Individuals with an overall score above five are categorized as poor sleepers. The Insomnia Severity Index (ISI) is a seven-item measure used to assess the perceived severity of clinically significant insomnia over a two-week period. Each item is rated on a 5-point Likert scale (0 = none to 4 = very many), and the total score is obtained by summing the seven items. Possible scores on the ISI range from 0 to 28, with higher scores indicating greater insomnia severity. Although the specific outcome indicators may vary across different studies, higher scores on the respective scales used indicate poorer sleep quality. Therefore, appropriate transformations can be applied to the statistical analysis (11).

### 3.6 Network meta-analysis

All p-values of indirect and direct comparisons between studies were tested for consistency and inconsistency. It was found that all p-values were more significant than 0.05, indicating that the consistency effect between studies was acceptable.

**Figure 6:**
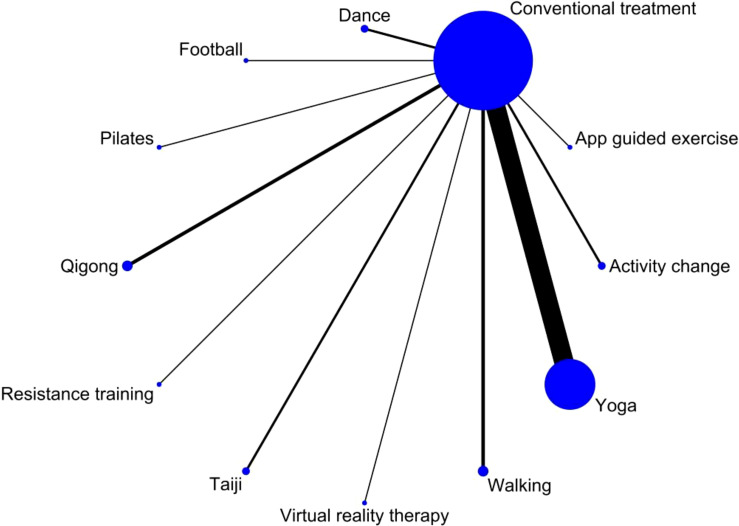
Network plot illustrating direct comparisons between exercise interventions and the non-exercise control group. Each node represents an exercise intervention, and each line represents a direct comparison from one or more studies included in the meta-analysis. Indirect comparisons between different exercise interventions are not included in this analysis.

The relationship between exercise therapy and a control group without exercise intervention is depicted in a network diagram. Each node in the diagram represents an intervention, with its size indicating the number of patients involved. The lines connecting the nodes represent direct relationships, and their thickness represents the number of studies directly compared. Based on the network diagram, yoga had the largest sample size among the exercise group interventions. On the other hand, Pilates, virtual reality exercises, soccer, resistance exercises, and software-guided exercises had relatively smaller sample sizes (**Figure 6**).

The network meta-analysis revealed that walking exercise was statistically significant in improving sleep disorders in BCS compared to conventional measures in the control group [SMD=-3.06, 95% CI=-5.89, -0.23] (**Table 3**). Additionally, walking exercise was found to be significantly superior to other exercise interventions. Among the various exercise interventions for improving sleep disorders in BCS, walking exercise had the highest SUCRA value (84.5%), followed by Pilates (83.5%), Qigong (64.8%), Tai Chi (55.3%), Yoga (55.0%), Software-guided exercise (46.3%), Dance (41.7%), Activity change exercise (38.5%), Resistance training (34.9%), Football (33.5%), and Virtual reality therapy (32.0%) (**Table 4**).

**Table 3:** Aggregated meta-analytic effect sizes for efficacy at posttreatment.

App guided exercise											
-0.83 (-6.44,4.79)	Walking										
-2.00 (-7.42,3.42)	-1.17 (-4.91,2.56)	Conventional treatment									
-2.47 (-8.07,3.14)	-1.64 (-5.63,2.36)	-0.46 (-4.17,3.25)	Football								
-2.68 (-7.63,2.28)	-1.85 (-4.87,1.17)	-0.68 (-3.31,1.96)	-0.21 (-3.20,2.78)	Qigong							
-3.00 (-9.33,3.33)	-2.17 (-7.13,2.79)	-1.00 (-5.74,3.74)	-0.53 (-5.48,4.41)	-0.32 (-4.52,3.88)	Resistance training						
-3.24 (-8.93,2.44)	-2.41 (-6.52,1.69)	-1.24 (-5.08,2.59)	-0.78 (-4.87,3.31)	-0.57 (-3.71,2.58)	-0.24 (-5.28,4.79)	Taiji					
-3.50 (-9.61,2.61)	-2.67 (-7.36,2.02)	-1.50 (-5.95,2.95)	-1.03 (-5.70,3.63)	-0.82 (-4.69,3.05)	-0.50 (-6.02,5.02)	-0.26 (-5.02,4.51)	Dance				
-3.40 (-8.94,2.13)	-2.57 (-6.47,1.32)	-1.40 (-5.01,2.21)	-0.94 (-4.81,2.94)	-0.72 (-3.59,2.14)	-0.40 (-5.27,4.46)	-0.16 (-4.15,3.84)	0.10 (-4.49,4.69)	Activity change			
-3.83 (-9.93,2.27)	-3.00 (-7.67,1.67)	-1.83 (-6.26,2.61)	-1.36 (-6.02,3.29)	-1.15 (-5.01,2.70)	-0.83 (-6.34,4.68)	-0.59 (-5.34,4.17)	-0.33 (-5.59,4.93)	-0.43 (-5.00,4.15)	Virtual reality therapy		
-4.11 (-10.94,2.72)	-3.28 (-8.87,2.31)	-2.11 (-7.50,3.28)	-1.64 (-7.22,3.93)	-1.43 (-6.36,3.49)	-1.11 (-7.41,5.19)	-0.87 (-6.52,4.79)	-0.61 (-6.70,5.48)	-0.71 (-6.21,4.80)	-0.28 (-6.36,5.80)	Pilates	
-3.60 (-8.45,1.25)	-2.77 (-5.61,0.07)	-1.60 (-4.03,0.83)	-1.13 (-3.94,1.67)	-0.92 (-1.95,0.11)	-0.60 (-4.67,3.47)	-0.36 (-3.33,2.61)	-0.10 (-3.83,3.63)	-0.20 (-2.87,2.48)	0.23 (-3.48,3.94)	0.51 (-4.31,5.33)	Yoga

**Table 4:** SUCRA value.

Treatm~t	SUCRA	PrBest	MeanRank
A	38.5	0.8	7.8
B	46.3	5.6	6.9
C	30.0	0.0	8.7
D	41.7	1.9	7.4
E	33.5	1.7	8.3
F	83.5	48.3	2.8
G	64.8	4.8	4.9
H	34.9	2.1	8.2
I	55.3	3.3	5.9
J	32.0	3.3	8.5
K	84.5	28.1	2.7
L	55.0	0.1	6.0

A: Activity change exercise.

B: Software-guided exercise.

C: Conventional treatment.

D: Dance.

E: Football.

F: Pilates.

G: Qigong.

H: Resistance training.

I: Tai Chi.

J: Virtual reality therapy.

K: Walking.

L: Yoga.

### 3.7 Publication bias test

**Figure 7:**
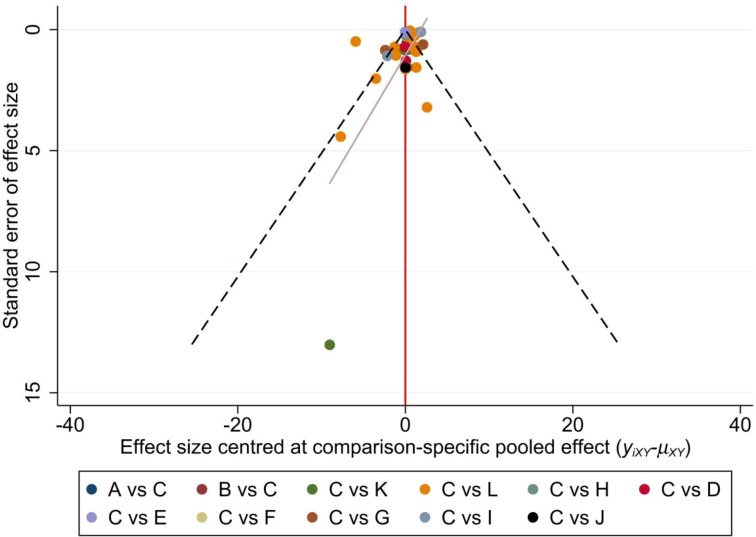
Funnel plot. A: Activity change exercise; B: Software-guided exercise; C: Conventional treatment; D: Dance; E: Football; F: Pilates; G: Qigong; H: Resistance training; I: Tai Chi; J: Virtual reality therapy; K: Walking; L: Yoga.

Sleep quality was chosen as the outcome measure for constructing a funnel plot. The funnel plot demonstrated that the distribution of scatter points on both sides of the red indicator line did not exhibit any noticeable asymmetry. Only one point fell outside the funnel plot, indicating a low likelihood of a small sample effect or publication bias in the study (**Figure 7**).

## 4 Discussion

In conclusion, 34 RCTs involving 3083 BCS were included in this study to explore the effects of various exercise regimens on sleep disorders. We found that walking exercise was the most recommended regimens for improving sleep quality in BCS. Our findings are credible due to the inclusion of large sample sizes and the application of rigorous screening methods in randomized controlled trials.

Sleep disturbance is a significant issue for BCS, ranking among the top five long-term problems they face. This problem persists both before and after cancer diagnosis and treatment. As breast cancer incidence rates continue to rise and mortality rates decrease, more and more BCS will experience sleep disturbance in the future. Several factors contribute to sleep disorders in these survivors, including physical and psychological changes resulting from diagnosis and treatment, such as age, physical activity, pain, hot flashes, night sweats, medication use, anxiety, and depression (7, 43, 44). It is widely recognized that pain negatively affects sleep quality, and the presence of sleep disorders can also heighten pain sensitivity. Mastectomy is often accompanied by significant pain, and neuropathy-induced stinging pain is a common source of discomfort for patients undergoing chemotherapy. The reported prevalence of neuropathic pain ranges from 11% to 80% in short-term survivors and 17% in long-term survivors of breast cancer (45). The physical and emotional effects of a cancer diagnosis and treatment frequently lead to increased anxiety, depression, fear of recurrence, and fatigue, all of which directly impact sleep quality (46). Additionally, rumination and worry can affect bedtime, nighttime awakenings, and the perception of poor sleep quality. It is worth noting that chemotherapy and steroid drugs, as well as vasomotor symptoms like hot flashes and night sweats, are associated with a higher incidence of sleep disturbance in BCS (47, 48). Based on our analysis and previous research, walking exercise shows promise in addressing the underlying causes of sleep disturbance and improving other symptoms in cancer patients.

Other exercise interventions, including resistance training, yoga, and tai chi, have demonstrated improvements in sleep quality, albeit to a lesser extent. This discrepancy may be attributed to factors such as the intensity, duration, and frequency of the exercise, as well as the levels of physical and mental relaxation achieved, and the extent of physical fatigue experienced post-exercise. The effectiveness of an exercise intervention might also depend on its ability to be consistently performed and tailored to individual needs, which walking readily allows.

A consensus statement from the International Multidisciplinary Roundtable Guidelines suggests that (49) cancer survivors can improve their sleep by engaging in moderate-intensity aerobic training, such as walking exercise, for 30 to 40 minutes three to four times per week, which aligns with the findings of our study. Walking exercise is a low-cost and easy-to-perform aerobic activity that may be a suitable option for BCS for several reasons: (1) It is a safe and easy-to-learn form of exercise that can be incorporated into daily activities. It is not influenced by factors like age, region, or education level, and the exercise duration, frequency, and intensity can be measured and adjusted using timers or mobile detection devices. (2) BCS often experience osteoporosis and joint pain due to the use of neurotoxic chemotherapy drugs and aromatase inhibitors (50). However, there have been no reports of side effects, such as fractures or falls, associated with walking exercise. This may explain why BCS are able to adhere to the exercise guidelines and improve sleep quality through increased physical exertion. (3) BCS can combine meditation with walking exercises. Walking exercise meditation not only enhances cardiometabolic health and basic vascular function but also helps alleviate side effects like insomnia and fatigue. (4) Compared to exercising in urban spaces, walking in natural environments can offer a range of physical health benefits (51–53), such as improved sleep, immune function, post-operative recovery, and pain relief. Therefore, incorporating walking as a form of exercise can provide a holistic approach to enhancing sleep for individuals with BCS.

Recognizing the high incidence and persistence of sleep disturbances in BCS is crucial for clinical diagnosis and intervention. Oncologists should also consider the association between sleep disturbances and related issues such as fatigue and depression. Personalized exercise prescription has gained significant importance in recent years due to mounting evidence suggesting that exercise can have a positive impact on sleep quality and the overall quality of life for BCS. Our meta-analysis indicates that a tailored walking program may be particularly beneficial for BCS experiencing sleep disorders, with varying effects observed across different regions and cancer stages. Specifically, walking exercise has shown to be advantageous for BCS and patients in clinical stages I to III in North America and Asian countries, suggesting a higher effectiveness in these areas. This disparity is influenced not only by regional factors such as altitude, climate, diet, and sports culture but also by the treatment received by patients at different clinical stages, their physical recovery, and the tolerance for exercise intensity. Consequently, clinical professionals should consider developing and continuously adjusting training plans that take into account the patient’s clinical stage, nationality, physical condition, and personal preferences. Such plans may involve increasing training duration, providing psychoeducation, and offering social support. While our study found that other exercise modalities such as Pilates and Qigong were not as effective as walking, they still had a positive impact on breast cancer patients with sleep disorders. Therefore, if a patient prefers alternative forms of exercise and they are generally safe, they should be encouraged to choose their preferred exercise modality. It is important to note that although exercise interventions for cancer patients are generally less intensive, high-risk patients, especially those with concomitant heart or lung disease, still require close monitoring.

Future studies should prioritize investigating the effects of walking and other forms of moderate to low-intensity exercise on post-cancer symptomatology in cancer patients. Additionally, research should explore the impact of various exercise modalities on sleep disorders across diverse countries, regions, and preoperative cancer classifications. This approach aims to equip clinicians with personalized exercise prescriptions tailored to patients’ unique regional contexts and disease trajectories. This research will contribute to enhancing exercise medicine treatment options for sleep disorders in BCS. Additionally, future research should aim to investigate the impact of different types of exercise on the sleep quality of BCS across various countries and stages of the disease. It is also important to explore the optimal timing, frequency, and intensity of exercise, as well as develop an evaluation system for objectively measuring sleep status to reduce subjective judgment errors.

## 5 Strengths and limitations

This systematic review is the first to specifically examine sleep disorders in BCS. It reviews the existing literature on the effects of exercise interventions on sleep quality in breast cancer patients. The included studies are not limited by country, race, or language. Additionally, this study conducted a comprehensive network meta-analysis to rank the effectiveness of different types of exercise in improving sleep disturbance among breast cancer patients. The findings of this study can serve as a valuable reference for both patients and healthcare providers.

In our network meta-analysis examining the effects of exercise interventions on the sleep quality of breast cancer survivors, several critical limitations were identified. These limitations, to a certain extent, compromise the statistical power and the broad applicability of our findings, thereby impacting their overall scientific merit. Despite the implementation of rigorous inclusion and exclusion criteria to mitigate heterogeneity, significant differences among the study participants in terms of sample size, the intensity of exercise interventions, and stages of breast cancer remained evident. Notably, specific exercise interventions such as Pilates, virtual reality training, football, resistance training, and software-guided physical activities had smaller sample sizes, which limited their statistical significance. In the study, these particular forms of exercise constituted a relatively minor proportion, with Yoga accounting for 50%, whereas Walking and Qigong made up only 8.82%, Dance 5.88%, and Pilates, Virtual Reality Therapy, Football, Resistance Training, and Software-guided Exercises each representing 2.94%. This disproportionate distribution could lead to an increase in statistical error, particularly when considering the unequal distribution of cancer stages among patients in various studies, with approximately 25% in stage I, 50% in stage II, 20% in stage III, and 5% in stage IV, potentially limiting the generalizability of the conclusions drawn from these findings.

In addition to the issues of sample size and heterogeneity, the general lack of long-term follow-up data is also a concern. Most studies reported short-term improvements in sleep quality following the intervention, without monitoring the long-term effects, which complicates the evaluation of the interventions’ sustainability. Concerning the potential for publication bias, although funnel plot analysis appears to suggest a more optimistic outlook, it does not fully address the disturbances caused by small study effects or selective publication, especially under the circumstance where positive results are more likely to be published, whereas negative findings tend to be overlooked.

The methods used to assess sleep quality also warrant attention; most relied on subjective tools such as the patient-reported Pittsburgh Sleep Quality Index. Despite their widespread applicability, these tools may introduce inherent reporting biases. To enhance the certainty and objectivity of outcomes, future research assessing the impact of intervention measures should increasingly incorporate objective assessment tools like polysomnography or actigraphy.

To bolster the scientific foundation and credibility of future research, conducting more high-quality randomized controlled trials is of utmost importance. This includes, but is not limited to, standardizing intervention measures, increasing sample sizes, and performing long-term follow-ups while employing more objective measurement tools. Through these efforts, we can gain a more comprehensive understanding of the potential effects of various exercise interventions on the sleep quality of breast cancer survivors, thereby offering stronger scientific support for clinical practices.

This study specifically aimed to assess the direct impact of different exercise interventions on sleep quality in breast cancer survivors by comparing each intervention to a non-exercise control group. Indirect comparisons and interactions between different exercise interventions were not within the scope of this analysis. Future research could explore these aspects to provide a more comprehensive understanding of the relative effectiveness of different exercise interventions.

## 6 Conclusion

In conclusion, our meta-analysis showed that walking exercise is effective in improving sleep disorders among BCS, compared to other forms of exercise. However, more clinical randomized controlled trials and controlled trials with larger cohorts and consistent intervention strategies might support or offer different opinions on the efficacy of these different exercises.

## Data Availability

The original contributions presented in the study are included in the article/supplementary material. Further inquiries can be directed to the corresponding author.
